# Effects of Rikkunshito (traditional Japanese medicine) on enteral feeding and the plasma ghrelin level in critically ill patients: a pilot study

**DOI:** 10.1186/s40560-014-0053-4

**Published:** 2014-09-02

**Authors:** Mineji Hayakawa, Yuichi Ono, Takeshi Wada, Yuichiro Yanagida, Atsushi Sawamura, Hiroshi Takeda, Satoshi Gando

**Affiliations:** Emergency and Critical Care Center, Hokkaido University Hospital, N14W5 Kita-ku, Sapporo, 060-8648 Japan; Gastroenterology and Hematology, Hokkaido University Graduate School of Medicine, Sapporo, 060-8638 Japan

**Keywords:** Enteral nutrition, Prokinetic drug, Gastroparesis, Complementary therapies, Metoclopramide

## Abstract

**Background:**

Rikkunshito is a traditional Japanese medicine that has been widely prescribed for patients with various gastrointestinal symptoms. Recently, the prokinetic effects of Rikkunshito in patients with a variety of diseases have attracted attention in Japan. The prokinetic effects of Rikkunshito are believed to result from an increase of active ghrelin, which is most abundant in the stomach and which has a gastrokinetic function. The aim of the present pilot study was to investigate the effects of Rikkunshito on intragastric enteral feeding and plasma ghrelin levels in critically ill patients.

**Methods:**

The study population consisted of critically ill patients who were projected to require intragastric tube feeding for more than 7 days. The patients were prospectively assigned to one of two treatment groups and were randomized to receive either Rikkunshito (2.5 g) or metoclopramide (10 mg) every 8 h. All patients received standard enteral nutrition. Patients in both groups were begun on intragastric tube feeding according to our institution’s feeding protocol.

**Results:**

All patients were undergoing mechanical ventilation at the time of enrollment. The portions of enteral nutrition provided to the target amount and the quantity of gastric discharge were not statistically significantly different between the two groups. The Rikkunshito group reached 50% of the target amount of enteral feeding significantly earlier than the metoclopramide group, although the proportion of patients in whom enteral feeding was successful did not differ significantly between the two groups. Patients in the Rikkunshito group showed significantly higher plasma levels of active ghrelin compared to those in the metoclopramide group.

**Conclusions:**

The administration of Rikkunshito increased the plasma level of active ghrelin, and induced prokinetic effects that were greater than those observed following treatment with metoclopramide in critically ill patients.

**Trial registration:**

UMIN00000356

## Background

Nutritional support is an important treatment for critically ill patients. Enteral support is preferred to parenteral support, particularly in patients with prolonged intensive care admissions [1]. However, gastrointestinal motility is frequently impaired in critically ill patients, and up to 50% of these patients are unable to tolerate enteral feeding [2].

Intolerance of intragastric enteral feeding is usually managed either by a change in the feeding route or by pharmacological interventions [3]. While various prokinetic drugs are available, few have been fully studied in critically ill patients. Metoclopramide and erythromycin have been studied in critical care settings [4]. Metoclopramide improves gastric motility in critically ill patients [4]. Erythromycin, a macrolide antibiotic, also acts as a motilin receptor agonist and stimulates gastric motility [3]. Several reports have indicated that erythromycin improves gastric emptying in critically ill patients [5–8].

Rikkunshito, a traditional Japanese medicine (Kampo), has been widely prescribed for patients with various gastrointestinal symptoms. Rikkunshito is extracted from a mixture of *Glycyrrhizae radix* (4.7%), *Zingiberis rhizoma* (2.3%), *Atractylodis lanceae rhizoma* (18.6%), *Zizyphi fructus* (9.3%), *Aurantii nobilis pericarpium* (9.3%), *Ginseng radix* (18.6%), *Pinelliae tuber* (18.6%), and *Hoelen* (18.6%). Recently, the prokinetic activity of Rikkunshito has attracted increased interest for the treatment of various diseases in Japan [9–14]. In patients with gastroesophageal reflux disease and functional dyspepsia, Rikkunshito improves clinical symptoms [9–12], gastric emptying [11], and esophageal acid clearance [9]. Additionally, in patients with persistent dyspepsia following gastrointestinal surgery, Rikkunshito improves both dyspeptic symptoms and gastric electrical activity [13]. Several flavonoids in Rikkunshito have serotonin 2B/2C receptor antagonist activity and accelerate active ghrelin release [15,16]. These clinical effects were related to an increase in plasma ghrelin levels following Rikkunshito administration [12,15,17]. However, no data are currently available on the use of either ghrelin or Rikkunshito to improve the tolerance of intragastric enteral feeding in critically ill patients [3].

This study aimed to investigate the effect of Rikkunshito on the tolerance of intragastric enteral feeding and plasma ghrelin levels in critically ill patients. This single-center, double-blind, randomized controlled trial is a pilot study to evaluate the utility of Rikkunshito in comparison with metoclopramide in the critical care setting.

## Methods

The present study was approved by the Institutional Review Board of Hokkaido University Hospital and was registered as UMIN00000356 with the University Hospital Medical Information Network (UMIN) Clinical Trials Registry. The study population consisted of critically ill patients who were projected to require intragastric tube feeding for more than 7 days. Informed consent was obtained from the family members of the patients. Patients were excluded from the study for the following reasons: (1) age less than 18 years, (2) contraindications to the use of Rikkunshito or metoclopramide, or (3) use of Rikkunshito or metoclopramide prior to inclusion in the present study.

After enrollment in the current study, patients were prospectively assigned to one of two treatment groups and were randomized to receive either Rikkunshito (TJ-43, Tsumura & Co., Tokyo, Japan) or metoclopramide (Primperan, Astellas Pharma Inc., Tokyo, Japan). All researchers involved in caring for the patients and collecting the data were blinded to the agents the patients received during the study period. In the Rikkunshito group, 2.5 g of Rikkunshito was administered intragastrically every 8 h via the feeding tube, while patients in the metoclopramide group received 10 mg of metoclopramide by the same route and with the same frequency. All patients received standard enteral nutrition (1 mL = 1 kcal) (Haine®, Otsuka Pharmaceutical Co., Ltd., Tokyo, Japan). Patients in both groups were begun on intragastric tube feeding according to our feeding protocol. The enteral feeding was initiated at a rate of 20 mL/h. Every 4 h, all residual gastric discharge was suctioned and measured. If the volume of gastric discharge was ≤100 mL, the full volume was returned to the patient, and the feeding rate was increased by 20 mL/h. The feeding rate was not increased over the target rate. If the volume of gastric discharge was >100 mL, only 100 mL was returned to the patient, and the enteral feeding was continued with no change in rate. When a volume greater than 100 mL of gastric discharge was observed on two consecutive measurements, the feeding rate was decreased by 20 mL/h. The target amount of calories and feeding rate were predetermined by referencing basal energy expenditure based on the Harris-Benedict equation [18]. The observation period was defined as either the time from the start of feeding until gastric feeding was no longer required or 10 days after enrollment in the current study. During the period of observation, the following treatments were prohibited: (1) the use of other prokinetic drugs (erythromycin, mosapride, domperidone, etc.) or (2) switch to intrajejunal tube feeding. Successful enteral feeding was defined as achievement of the target amount on two successive days. Achievement of 50% of the target amount of enteral feeding was defined as achieving 50% of the target amount of enteral feeding on two successive days.

At the beginning and end of the observation period, blood samples were collected from the patients in tubes containing aprotinin and EDTA-2Na (LSI Medience Corporation, Tokyo, Japan). The blood samples were immediately centrifuged at 4°C. Plasma was acidified with 1 mol/L HCl (1/10 volume) and stored at −80°C until measurement of ghrelin levels. The levels of active ghrelin and desacyl ghrelin (inactive ghrelin) were measured with commercial enzyme-linked immunosorbent assay (ELISA) kits (Active Ghrelin ELISA Kit and Desacyl Ghrelin ELISA Kit, SCETI KK, Tokyo, Japan). The primary endpoint of the study was the success rate of the enteral feeding. The secondary endpoints were the changes in the plasma levels of active ghrelin and desacyl ghrelin.

All measurements are expressed as the mean ± standard deviation (SD). The SPSS 15.0J statistical software package (SPSS Inc., Chicago, IL, USA) was used for all statistical analyses. Comparisons between the two groups were made using Student’s *t-*test, the chi-square test, or two-way repeated measures analysis of variance (ANOVA). The Kaplan-Meier method and the log-rank test were used to compare the success rates of enteral feeding between the two groups. A value of *P* < 0.05 was considered to be statistically significant.

## Results

**Figure 1:**
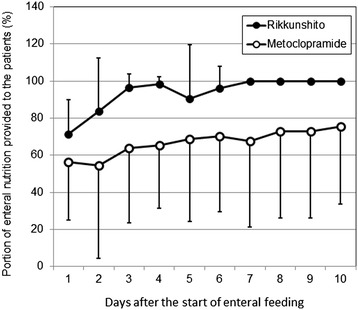
**The portion of enteral nutrition provided to the patients.** No differences were observed between the two groups during the observation period. *Open circle*, metoclopramide group; *closed circle*, Rikkunshito group. *Error bars* show standard deviation (SD).

**Figure 2:**
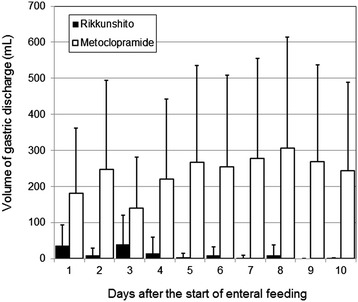
**The volume of gastric discharge.** No differences were observed between the two groups during the observation period. *Open bar*, metoclopramide group; *closed bar*, Rikkunshito group. *Error bars* show standard deviation (SD).

**Figure 3:**
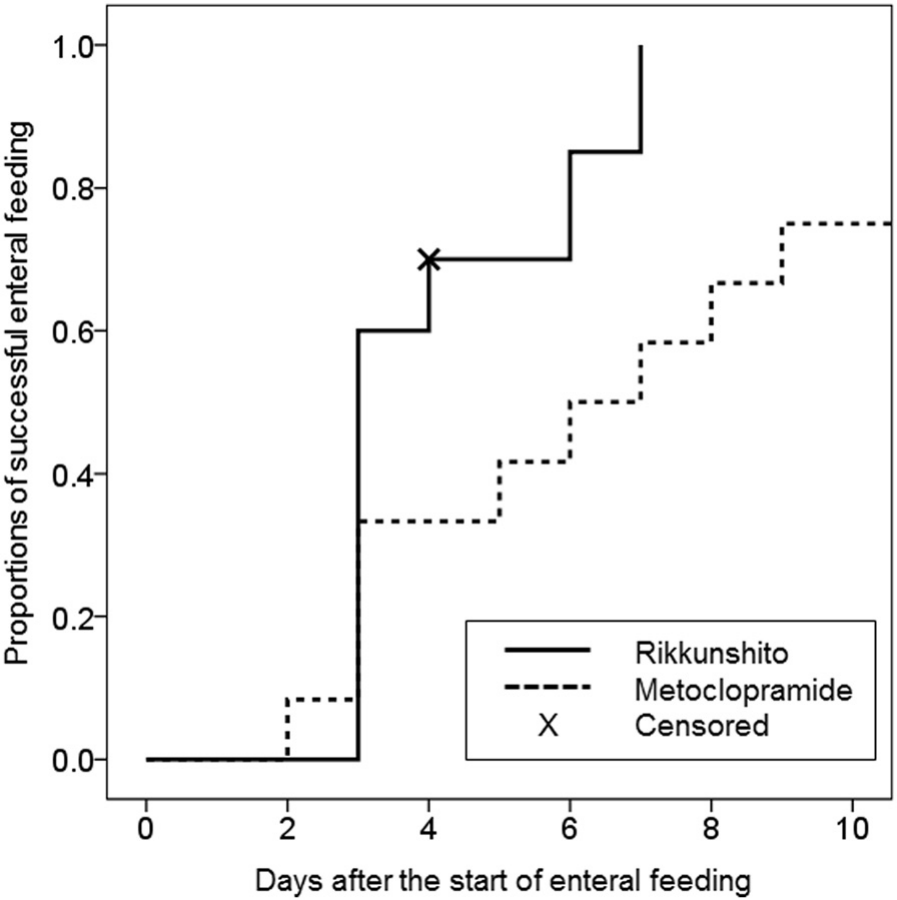
**The proportions of successful enteral feeding.** No differences were observed between the two groups during the observation period.

**Figure 4:**
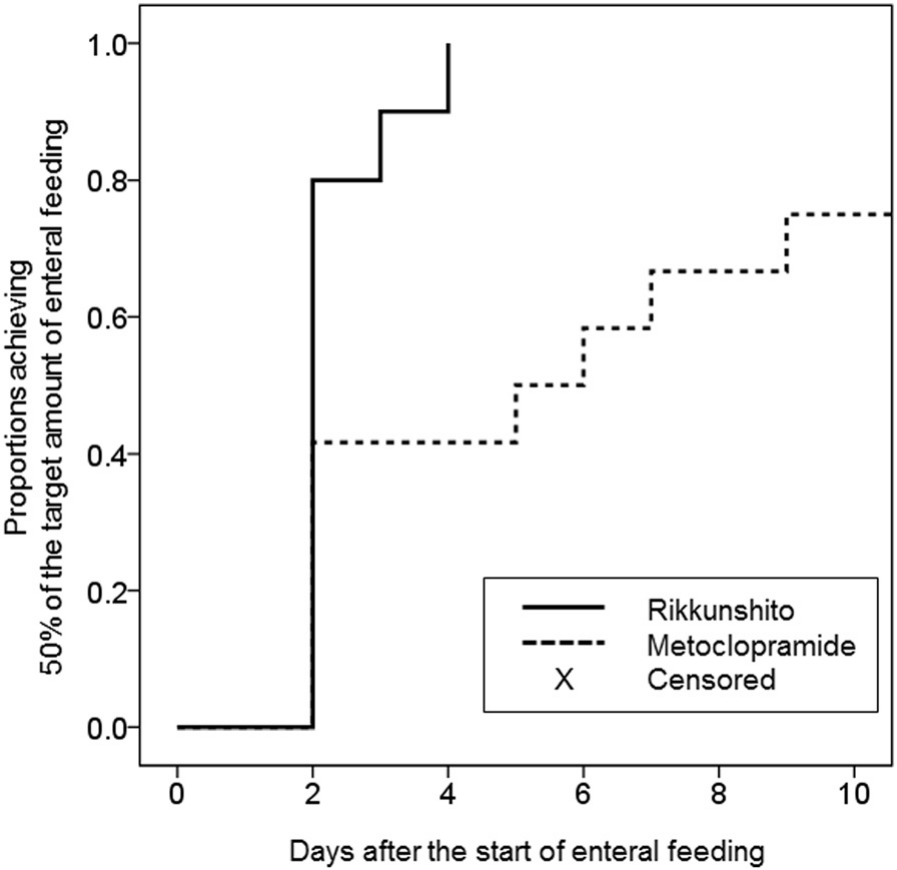
**The proportions achieving 50% of the target amount of enteral feeding.** The Rikkunshito group reached 50% of the target amount of enteral feeding significantly earlier than the metoclopramide group (*P* = 0.004).

Twenty-three patients were enrolled in the current study. All patients were undergoing mechanical ventilation at the time of enrollment. Thirteen patients were assigned to the metoclopramide group, and 10 patients were assigned to the Rikkunshito group. The characteristics of the patients in the two groups were well matched (Table 1). Neither the portions of enteral nutrition provided to the patients (Figure 1) nor the quantity of gastric discharge (Figure 2) differed significantly between the two groups. The proportion of patients in whom enteral feeding was successful also did not significantly differ between the two groups (Figure 3). However, the Rikkunshito group reached 50% of the target amount of enteral feeding significantly earlier than the metoclopramide group (*P* = 0.004) (Figure 4). No complications related to the enteral feeding were observed in either group. The plasma levels of active ghrelin and desacyl ghrelin are presented in Table 2. The increase in the active ghrelin concentration following treatment was larger in the Rikkunshito group than in the metoclopramide group (*P* = 0.023). The changes in the desacyl ghrelin concentrations did not significantly differ between the two groups during the observation period.

**Table 1:** **Characteristics of the patients**

	**Metoclopramide**	**Rikkunshito**	***P***
	***n*** **= 13**	***n*** **= 10**	
Age (years)	75 ± 11	70 ± 13	0.289
Sex (male/female)	8/5	5/5	0.685
Height (cm)	163 ± 9	162 ± 9	0.841
Weight (kg)	58.8 ± 10.8	60.4 ± 12.1	0.770
Body surface area (m^2^)	1.63 ± 0.16	1.64 ± 0.20	0.914
APACHE II score	28 ± 6	29 ± 10	0.774
Reason for admission to the ICU			
Post-cardiac arrest	5	4	
Trauma	3	2	
Cerebral infarction/hemorrhage	0	3	0.336
Burn	2	0	
Others	3	1	
Days of enrollment after admission to the ICU	3.5 ± 1.5	3.0 ± 2.3	0.563
C-reactive protein	8.3 ± 7.5	5.6 ± 6.7	0.385
Basal energy expenditure (kcal/day)	1,219 ± 161	1,255 ± 239	0.703
Target volume of enteral feeding (mL/h)	46.7 ± 7.8	51.0 ± 12.9	0.341
Observational period (day)	9.3 ± 2.2	9.8 ± 0.6	0.430

*APACHE* Acute Physiology and Chronic Health Evaluation, *ICU* intensive care unit.

**Table 2:** **Plasma levels of ghrelin**

	**Metoclopramide**	**Rikkunshito**	***P***
	***n*** **= 13**	***n*** **= 10**	
Active ghrelin (fmol/mL)			
Pre-treatment (day 0)	20.8 ± 17.1	29.2 ± 18.0	0.023
Post-treatment (day 10)	32.3 ± 37.9	60.4 ± 56.8	
Desacyl ghrelin (fmol/mL)			
Pre-treatment (day 0)	62.5 ± 27.5	77.9 ± 44.4	0.784
Post-treatment (day 10)	115.3 ± 63.8	122.8 ± 80.6	

## Discussion

The present pilot study is the first randomized controlled trial to evaluate the utility of Rikkunshito for enteral feeding in the critical care setting. The portions of enteral nutrition provided to the target amount and the quantity of gastric discharge did not significantly differ between the two groups. The Rikkunshito group reached 50% of the target amount of enteral feeding significantly earlier than the metoclopramide group, although the proportion of patients in whom enteral feeding was successful did not differ significantly between the two groups.

Significant increases in the plasma active ghrelin levels were observed in the Rikkunshito group in comparison to the metoclopramide group. Previous studies have found that metoclopramide led to improvement in gastric emptying in critically ill patients with an intolerance of enteral feeding, compared to placebo [4,19]. The present study, however, included patients with and without an intolerance of normal enteral feeding, because patients were enrolled at the beginning of the enteral feeding period. Moreover, the present pilot study had a small sample size. Therefore, this study may have been underpowered to detect differences between Rikkunshito and metoclopramide in the proportion of successful enteral feeding.

Ghrelin, a growth hormone-releasing acylated peptide, is a ligand for the growth hormone secretagogue receptor (GHS-R) in stomach tissue [20]. However, ghrelin and motilin, as well as GHS-R and the motilin receptor, are structurally related [21]. Furthermore, ghrelin is most abundant in the stomach, while GHS-R is present in both the stomach and other organs [21]. Therefore, ghrelin, like motilin, has been concluded to have a gastrokinetic function [3,21]. In functional dyspepsia, the plasma active ghrelin levels are related to gastric emptying and clinical symptoms [12,22]. In diabetic gastroparesis patients, intravenous ghrelin administration enhances gastric emptying [23]. The prokinetic effects of active ghrelin include an induction of premature phase III activity and a prolonged increase in proximal gastric tone [24]. These are direct effects, not mediated through the release of other gastrointestinal hormones [24]. In this study, Rikkunshito administration both accelerated active ghrelin release and improved gastrokinetic function.

Although metoclopramide is a traditional prokinetic drug, its effects on the release of active ghrelin are unclear. Metoclopramide acts as a dopamine receptor antagonist, a serotonin antagonist (serotonin 3 receptor), and a serotonin partial agonist (serotonin 4 receptor) [25]. The drug leads to the release of acetylcholine from gut neurons, antagonizing the inhibitory effects of dopamine on gastrointestinal motility [3]. A previous study indicated that the cholinergic system controlled the release of ghrelin and that acetylcholine increased the plasma concentration of active ghrelin [26,27]. Metoclopramide was not found to activate the secretion of ghrelin in an animal study [28]; therefore, it seems unlikely that the prokinetic effects of metoclopramide observed in the current study are related to ghrelin secretion.

## Conclusions

In the present study, the administration of Rikkunshito led to increased plasma levels of active ghrelin and induced prokinetic effects that were greater than those observed following treatment with metoclopramide in critically ill patients. Although Rikkunshito appears to be an effective stimulator of active ghrelin secretion and improves gastrokinetic function in the critical care setting, further investigations with a larger sample size will be required to clarify the effects of Rikkunshito in patients with an intolerance of enteral feeding.

## References

[1] Martindale RG, McClave SA, Vanek VW, McCarthy M, Roberts P, Taylor B, Ochoa JB, Napolitano L, Cresci G, American College of Critical Care Medicine; A.S.P.E.N. Board of Directors (2009). Guidelines for the provision and assessment of nutrition support therapy in the adult critically ill patient: Society of Critical Care Medicine and American Society for Parenteral and Enteral Nutrition: Executive Summary. Crit Care Med.

[2] Mentec H, Dupont H, Bocchetti M, Cani P, Ponche F, Bleichner G (2001). Upper digestive intolerance during enteral nutrition in critically ill patients: frequency, risk factors, and complications. Crit Care Med.

[3] Deane A, Chapman MJ, Fraser RJ, Bryant LK, Burgstad C, Nguyen NQ (2007). Mechanisms underlying feed intolerance in the critically ill: implications for treatment. World J Gastroenterol.

[4] Jooste CA, Mustoe J, Collee G (1999). Metoclopramide improves gastric motility in critically ill patients. Intensive Care Med.

[5] Berne JD, Norwood SH, McAuley CE, Vallina VL, Villareal D, Weston J, McClarty J (2002). Erythromycin reduces delayed gastric emptying in critically ill trauma patients: a randomized, controlled trial. J Trauma.

[6] Dive A, Miesse C, Galanti L, Jamart J, Evrard P, Gonzalez M, Installé E (1995). Effect of erythromycin on gastric motility in mechanically ventilated critically ill patients: a double-blind, randomized, placebo-controlled study. Crit Care Med.

[7] Chapman MJ, Fraser RJ, Kluger MT, Buist MD, De Nichilo DJ (2000). Erythromycin improves gastric emptying in critically ill patients intolerant of nasogastric feeding. Crit Care Med.

[8] Reignier J, Bensaid S, Perrin-Gachadoat D, Burdin M, Boiteau R, Tenaillon A (2002). Erythromycin and early enteral nutrition in mechanically ventilated patients. Crit Care Med.

[9] Kawahara H, Kubota A, Hasegawa T, Okuyama H, Ueno T, Ida S, Fukuzawa M (2007). Effects of rikkunshito on the clinical symptoms and esophageal acid exposure in children with symptomatic gastroesophageal reflux. Pediatr Surg Int.

[10] Tominaga K, Iwakiri R, Fujimoto K, Fujiwara Y, Tanaka M, Shimoyama Y, Umegaki E, Higuchi K, Kusano M, Arakawa T, GERD 4 Study Group (2012). Rikkunshito improves symptoms in PPI-refractory GERD patients: a prospective, randomized, multicenter trial in Japan. J Gastroenterol.

[11] Kusunoki H, Haruma K, Hata J, Ishii M, Kamada T, Yamashita N, Honda K, Inoue K, Imamura H, Manabe N, Shiotani A, Tsunoda T (2010). Efficacy of Rikkunshito, a traditional Japanese medicine (Kampo), in treating functional dyspepsia. Intern Med.

[12] Arai M, Matsumura T, Tsuchiya N, Sadakane C, Inami R, Suzuki T, Yoshikawa M, Imazeki F, Yokosuka O (2012). Rikkunshito improves the symptoms in patients with functional dyspepsia, accompanied by an increase in the level of plasma ghrelin. Hepatogastroenterology.

[13] Yagi M, Homma S, Kubota M, Iinuma Y, Kanada S, Kinoshita Y, Ohtaki M, Yamazaki S, Murata H (2004). The herbal medicine Rikkunshi-to stimulates and coordinates the gastric myoelectric activity in post-operative dyspeptic children after gastrointestinal surgery. Pediatr Surg Int.

[14] Kawahara H, Mitani Y, Nomura M, Nose K, Yoneda A, Hasegawa T, Kubota A, Fukuzawa M (2009). Impact of rikkunshito, an herbal medicine, on delayed gastric emptying in profoundly handicapped patients. Pediatr Surg Int.

[15] Takeda H, Sadakane C, Hattori T, Katsurada T, Ohkawara T, Nagai K, Asaka M (2008). Rikkunshito, an herbal medicine, suppresses cisplatin-induced anorexia in rats via 5-HT2 receptor antagonism. Gastroenterology.

[16] Kido T, Nakai Y, Kase Y, Sakakibara I, Nomura M, Takeda S, Aburada M (2005). Effects of rikkunshi-to, a traditional Japanese medicine, on the delay of gastric emptying induced by N(G)-nitro-L-arginine. J Pharmacol Sci.

[17] Matsumura T, Arai M, Yonemitsu Y, Maruoka D, Tanaka T, Suzuki T, Yoshikawa M, Imazeki F, Yokosuka O (2010). The traditional Japanese medicine Rikkunshito increases the plasma level of ghrelin in humans and mice. J Gastroenterol.

[18] Harris JA, Benedict FG (1918). A biometric study of human basal metabolism. Proc Natl Acad Sci USA.

[19] MacLaren R, Kuhl DA, Gervasio JM, Brown RO, Dickerson RN, Livingston TN, Swift K, Headley S, Kudsk KA, Lima JJ (2000). Sequential single doses of cisapride, erythromycin, and metoclopramide in critically ill patients intolerant to enteral nutrition: a randomized, placebo-controlled, crossover study. Crit Care Med.

[20] Kojima M, Hosoda H, Date Y, Nakazato M, Matsuo H, Kangawa K (1999). Ghrelin is a growth-hormone-releasing acylated peptide from stomach. Nature.

[21] Peeters TL (2005). Ghrelin: a new player in the control of gastrointestinal functions. Gut.

[22] Shindo T, Futagami S, Hiratsuka T, Horie A, Hamamoto T, Ueki N, Kusunoki M, Miyake K, Gudis K, Tsukui T, Iwakiri K, Sakamoto C (2009). Comparison of gastric emptying and plasma ghrelin levels in patients with functional dyspepsia and non-erosive reflux disease. Digestion.

[23] Murray CD, Martin NM, Patterson M, Taylor SA, Ghatei MA, Kamm MA, Johnston C, Bloom SR, Emmanuel AV (2005). Ghrelin enhances gastric emptying in diabetic gastroparesis: a double blind, placebo controlled, crossover study. Gut.

[24] Tack J, Depoortere I, Bisschops R, Delporte C, Coulie B, Meulemans A, Janssens J, Peeters T (2006). Influence of ghrelin on interdigestive gastrointestinal motility in humans. Gut.

[25] Rizzi CA, Mierau J, Ladinsky H (1997). Regulation of plasma aldosterone levels by metoclopramide: a reappraisal of its mechanism from dopaminergic antagonism to serotonergic agonism. Neuropharmacology.

[26] Maier C, Schaller G, Buranyi B, Nowotny P, Geyer G, Wolzt M, Luger A (2004). The cholinergic system controls ghrelin release and ghrelin-induced growth hormone release in humans. J Clin Endocrinol Metab.

[27] Shrestha YB, Wickwire K, Giraudo SQ (2009). Direct effects of nutrients, acetylcholine, CCK, and insulin on ghrelin release from the isolated stomachs of rats. Peptides.

[28] Sugino T, Yamaura J, Yamagishi M, Kurose Y, Kojima M, Kangawa K, Hasegawa Y, Terashima Y (2003). Involvement of cholinergic neurons in the regulation of the ghrelin secretory response to feeding in sheep. Biochem Biophys Res Commun.

